# Comparison of Computer Vision and Photogrammetric Approaches for Epipolar Resampling of Image Sequence

**DOI:** 10.3390/s16030412

**Published:** 2016-03-22

**Authors:** Jae-In Kim, Taejung Kim

**Affiliations:** Department of Geoinformatic Engineering, Inha University, 100 Inharo, Nam-gu Incheon 22212, Korea; jikim3124@inha.edu

**Keywords:** epipolar resampling, image rectification, Bayesian approach, stereo image sequence

## Abstract

Epipolar resampling is the procedure of eliminating vertical disparity between stereo images. Due to its importance, many methods have been developed in the computer vision and photogrammetry field. However, we argue that epipolar resampling of image sequences, instead of a single pair, has not been studied thoroughly. In this paper, we compare epipolar resampling methods developed in both fields for handling image sequences. Firstly we briefly review the uncalibrated and calibrated epipolar resampling methods developed in computer vision and photogrammetric epipolar resampling methods. While it is well known that epipolar resampling methods developed in computer vision and in photogrammetry are mathematically identical, we also point out differences in parameter estimation between them. Secondly, we tested representative resampling methods in both fields and performed an analysis. We showed that for epipolar resampling of a single image pair all uncalibrated and photogrammetric methods tested could be used. More importantly, we also showed that, for image sequences, all methods tested, except the photogrammetric Bayesian method, showed significant variations in epipolar resampling performance. Our results indicate that the Bayesian method is favorable for epipolar resampling of image sequences.

## 1. Introduction

Epipolar resampling is the procedure of eliminating *Y* parallax, or vertical disparity, between a stereo image pair. This procedure is critical both for stereo image processing and for three-dimensional (3D) content generation. For stereo image processing, this can convert two-dimensional (2D) correspondence search problems into one-dimensional (1D) ones and hence enhance automated depth map generation [1,2,3]. For 3D content generation, this can eliminate visual fatigue and produce high quality 3D perception [4]. In addition, this can enhance the processing of various stereovision systems such as mobile robots or smart vehicles.

Epipolar resampling has been studied extensively in the fields of computer vision and photogrammetry. In computer vision, epipolar resampling is achieved by a homographic transformation for sending epipoles of original images to infinity [5,6,7,8,9,10,11]. Depending on how the homography is estimated, epipolar resampling methods can be classified into two approaches: uncalibrated and calibrated cases. The uncalibrated approach estimates the homography from the fundamental matrix, which is determined using tie-points between a stereo image pair. The calibrated approach estimates the homography from known intrinsic and extrinsic parameters of stereo images. Typically, these parameters are acquired by a stereo calibration method using calibration patterns [12,13].

In photogrammetry, epipolar resampling is performed by a perspective transformation that aligns epipolar lines with horizontal image lines. This transformation is defined by relative orientation parameters between the two images. Collinear or coplanar equations are used to estimate the parameters mathematically [14,15,16,17]. In a typical photogrammetric application, intrinsic parameters are assumed to be known. Relative orientation parameters, which are extrinsic parameters of the right image with respect to the left image frame, are estimated by tie-points.

Epipolar resampling developed in each field is serving its own purpose and application. In computer vision, for example, epipolar resampling is used for robot vision and fast image processing [18,19,20]. In photogrammetry, it is mainly used for precise image rectification for stereo plotters [14,21,22]. For serving new and challenging applications, there is an increasing need for merging techniques developed in computer vision and photogrammetry. However, evaluation of epipolar resampling methods developed from each field using a common dataset, comparison of their performances and understanding of their differences in theoretical and practical perspectives are missing.

In this paper, we aim to compare epipolar resampling methods developed in computer vision and photogrammetry. In particular, we aim to apply epipolar resampling not for rectifying one stereo pair, but for rectifying a sequence of stereo images. We argue that most previous investigations were for epipolar resampling of a single image pair and that epipolar resampling of image sequences has not been studied thoroughly. Here we focus on epipolar resampling of image sequences with an intension of stereo applications where two cameras are installed at separate platforms and move independently.

We will first review the general principles and formulations of epipolar resampling developed in the computer vision field. We will then review the epipolar resampling developed in photogrammetry. We will re-confirm the well-known principle that epipolar resampling methods developed in computer vision and in photogrammetry are mathematically identical. We will also point out practical differences between them. We will then explain image sequences and the representative epipolar resampling methods used for tests. Finally, we report and compare their performance over a single image pair and over image sequences.

## 2. Epipolar Resampling in Computer Vision

**Figure 1:**
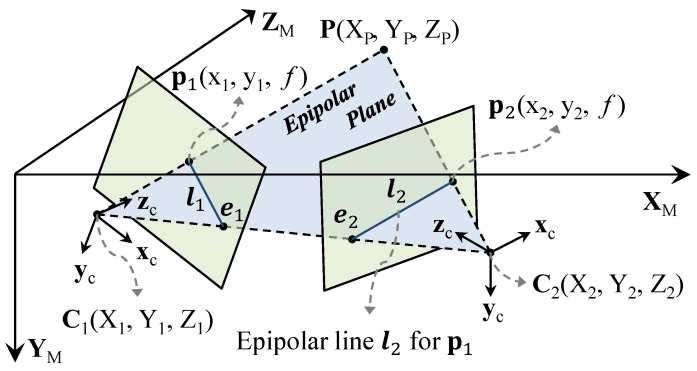
Epipolar geometry of stereo image.

Epipolar geometry between a stereo pair is explained in Figure 1. The two perspective centers ($C_{1}$ and $C_{2}$ in the figure) and a ground point P define an epipolar plane. Epipolar lines $l_{1}$ and $l_{2}$ are the intersection between the epipolar plane and the left and right image planes, respectively. Epipoles $e_{1}$ and $e_{2}$ are the intersections between the line connecting the two perspective centers and the left and right image planes.

For perspective images, any corresponding left and right image points $q_{1}$ and $q_{2}$ satisfy the following matrix equation [23]:

(1) $$q_{2}^{T}Fq_{1}=0$$

where $q_{1}\left(u_{1},v_{1},1\right)$ and $q_{2}\left(u_{2},v_{2},1\right)$ are homogenous image coordinates of the left and right image points ($p_{1}$ and $p_{2}$) and F the fundamental matrix. Epipolar lines $l_{1}$ and $l_{2}$ and epipoles $e_{1}$ and $e_{2}$ can be found by F:

(2) $$l_{1}=F^{T}q_{2},\;l_{2}=Fq_{1},\;Fe_{1}=0,\;F^{T}e_{2}=0$$

Epipolar resampling can be achieved by a homographic transformation of mapping the epipole of the original image to a point at infinity. The homography in the case of the uncalibrated approach can be determined from the fundamental matrix as

(3) H=GAT

where T is a translation taking the principal point ($c_{x},c_{y}$) of the image to the origin of the coordinate system, A is a rotation about the origin taking the epipole to a point ${\left(k,0,1\right)}^{T}$ on the *x-*axis, and G is a transformation taking the moved epipole to the point at infinity ${\left(k,0,0\right)}^{T}$ as shown below [8].

(4) $$T=\left[\begin{array}{ccc} 1 & 0 & -c_{x}\\ 0 & 1 & -c_{y}\\ 0 & 0 & 1 \end{array}\right],\;G=\left[\begin{array}{ccc} 1 & 0 & 0\\ 0 & 1 & 0\\ -1/k & 0 & 1 \end{array}\right]$$

Once the homographies are calculated, then optimization is generally carried out to align corresponding epipolar lines between stereo images [7,9,24].

Under the uncalibrated approach, the fundamental matrix can be estimated from tie-points without any additional information. Therefore, this approach enables automation over the whole process and the use of images from an unknown source. There are many studies regarding the reliable estimation of the fundamental matrix and the reliable reconstruction of epipolar geometry. Among them, included were Hartley’s normalized eight-point algorithm [25], and algorithms that apply additional constraints such as algebraic minimization [26], minimization of epipolar distance [8,24], and other geometric cost functions [27,28,29].

The uncalibrated approach may, however, be sensitive to tie-point noise and prone to image distortion as the transformation is not based on physical geometry between the two images. Fusiello and Irsara [30] tried to overcome these problems by proposing a new uncalibrated approach called quasi-Euclidean epipolar rectification. They estimated stereo geometry using non-linear equations by minimizing geometric re-projection errors.

In the calibrated approach, intrinsic and extrinsic parameters for the left and right images are estimated separately by the stereo calibration method using dedicated calibration patterns [12,13,31,32]. Extrinsic parameters are used to estimate the relative geometric relationship between the two images. The homography for epipolar resampling is then defined by the relative geometric relationship.

Assuming $R_{1}$ and $R_{2}$ are 3 × 3 rotation matrices containing rotational extrinsic parameters of the left and right cameras and $C_{1}$ and $C_{2}$ are 3 × 1 translation matrices containing extrinsic parameters related to the location of the perspective center, the relative rotation R and translation B between two images are defined as below [33]:

(5) $$R=R_{2}R_{1}^{T}=\left[\begin{array}{ccc} r_{11} & r_{12} & r_{13}\\ r_{21} & r_{22} & r_{23}\\ r_{31} & r_{32} & r_{33} \end{array}\right],\;B=R_{1}\left(C_{2}-C_{1}\right)=\left[\begin{array}{c} b_{x}\\ b_{y}\\ b_{z} \end{array}\right]$$

The homography, $H_{1}$ for the left and $H_{2}$ for the right image, can be estimated by two rotations. The first rotation, $R_{\text{half}}^{1}$ and $R_{\text{half}}^{2}$, is distributed from the relative rotation R to make the overlap between the two images reach maximum. The second rotation $R_{\text{rect}}$ transforms the epipole to infinity using the baseline vector B:

(6) $$H_{1}=R_{\text{rect}}R_{\text{half}}^{1},\;H_{2}=R_{\text{rect}}R_{\text{half}}^{2}$$

where $R=R_{\text{half}}^{1}{\left(R_{\text{half}}^{2}\right)}^{T}$, $R_{\text{rect}}={\left[\begin{array}{ccc} m_{1}^{T} & m_{2}^{T} & m_{3}^{T} \end{array}\right]}^{T}$ and $m_{1}$, $m_{2}$ and $m_{3}$ are defined as

(7) $$m_{1}=\frac{m}{\left||m|\right|},\;m_{2}=\frac{{\left[\begin{array}{ccc} -b_{y} & b_{x} & 0 \end{array}\right]}^{T}}{\sqrt{b_{x}^{2}+b_{y}^{2}}},\;m_{3}=m_{1}\times m_{2}$$

As mentioned above, while the calibrated approach guarantees minimized image distortion and high accuracy, it requires a prior calibration process. Accordingly, this approach potentially involves a limitation in terms of availability.

## 3. Photogrammetric Epipolar Resampling

In this section, we review the epipolar resampling developed in photogrammetry. While its mathematical formulation is well reported [17], we re-formulate photogrammetric camera models here in order to compare them more directly with the ones developed in computer vision. In photogrammetry, the geometric relationship between a ground point P and its left and right image points, $q_{1}$ and $q_{2}$, is explained by the collinear equation below:

(8) $$q_{1}=M_{1}p_{1}=\lambda_{1}M_{1}R_{1}P_{1},\;q_{2}=M_{2}p_{2}=\lambda_{2}M_{2}R_{2}P_{2}$$

where λ is a scale factor, $R_{1}$ and $R_{2}$ are 3 × 3 rotation matrices defined as before. $P_{1}=\vec{C_{1}P}$, $P_{2}=\vec{C_{2}P}$, and $p_{1}\left(x_{1},y_{1},f_{1}\right)$ and $p_{2}\left(x_{2},y_{1},f_{2}\right)$ are look vectors of the left and right camera frames from the perspective center to projection point. $M_{1}$ and $M_{2}$ are camera matrices of the left and right images for converting camera coordinates to image coordinates as follows:

(9) $$M_{1}=\left[\begin{array}{ccc} f_{1} & 0 & c_{x_{1}}\\ 0 & f_{1} & c_{y_{1}}\\ 0 & 0 & 1 \end{array}\right],\;M_{2}=\left[\begin{array}{ccc} f_{2} & 0 & c_{x_{2}}\\ 0 & f_{2} & c_{y_{2}}\\ 0 & 0 & 1 \end{array}\right]$$

where $c_{x}$ and $c_{y}$ are defined as Equation (4) and fis the focal length of the camera.

The geometric relationship between the stereo images can be explained by the condition that the baseline vector $S=\vec{C_{1}C_{2}}$, the left look vector $P_{1}$ and the right look vector $P_{2}$ are coplanar. This coplanar condition can be expressed by the scalar triple product among the three vectors as below.

(10) $$P_{2}^{T}\cdot \left[S\times P_{1}\right]=P_{2}^{T}{\left[S\right]}_{\times }P_{1}=0,\;{\left[S\right]}_{\times }=\left[\begin{array}{ccc} 0 & -s_{z} & s_{y}\\ s_{z} & 0 & -s_{x}\\ -s_{y} & s_{x} & 0 \end{array}\right]$$

Taking the left camera axes as the reference frame, *i.e.*, $R_{1}=I_{3\times 3}$ and $C_{1}=0_{3\times 1}$, the above coplanar equation can be re-written in the form of the fundamental matrix as shown below:

(11) $$P_{2}^{T}{\left[S\right]}_{\times }P_{1}=\;p_{2}^{T}R{\left[B\right]}_{\times }p_{1}=0$$

(12) $$q_{2}^{T}{\left(M_{2}^{-1}\right)}^{T}R{\left[B\right]}_{\times }M_{1}^{-1}q_{1}=q_{2}^{T}Fq_{1}=0$$

where R and B are the relative rotation and translation of the right camera with respect to the left image frame [17]. As shown in Equation (12), it is well known that the coplanar equation used in photogrammetry is mathematically identical to the fundamental matrix equation used in computer vision. The main difference in the uncalibrated and photogrammetric approaches is that the former estimates the whole eight parameters of the fundamental matrix from tie-points whereas the latter estimates relative rotational and translational parameters.

**Figure 2:**
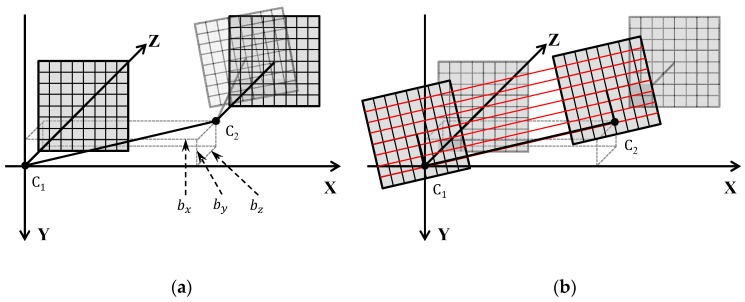
Procedure of epipolar transformation based on dependent relative orientation. (**a**) transformation for reprojecting the right image plane parallel to reference frame, (**b**) transformation for reprojecting two image planes to align with the baseline.

Epipolar resampling is carried out by two perspective transformations derived from the extrinsic parameters [17,34]. First, the right image plane is re-projected parallel to the reference frame (left camera axes) using the rotation angles of the right camera as shown in Figure 2a. Secondly, the two image planes are then re-projected to align with the baseline connecting two perspective centers, and to remove *Y* parallaxes as shown in Figure 2b.

These two perspective transformations can also be expressed by homography as

(13) $$H_{1}=R_{\text{base}}R_{1}^{T},\;H_{2}=R_{\text{base}}R_{2}^{T}$$

where $R_{\text{base}}$ is the second perspective transformation to align with respect to the baseline and is defined from the rotation angles of the baseline for *X*, *Y* and *Z* axes as

(14) $$R_{\text{base}}=R_{\Omega }R_{\Phi }R_{Κ}=\left[\begin{array}{ccc} 1 & 0 & 0\\ 0 & \text{cos}\Omega & \sin\Omega \\ 0 & -\sin\Omega & \text{cos}\Omega \end{array}\right]\left[\begin{array}{ccc} \text{cos}\Phi & 0 & -\sin\Phi \\ 0 & 1 & 0\\ \sin\Phi & 0 & \text{cos}\Phi \end{array}\right]\left[\begin{array}{ccc} \text{cos}Κ & \sin Κ & 0\\ -\sin Κ & \text{cos}Κ & 0\\ 0 & 0 & 1 \end{array}\right]$$

(15) $$\Omega =\frac{\omega_{1}+\omega_{2}}{2},\;\Phi =-{\text{tan}}^{-1}\frac{b_{z}}{\sqrt{b_{x}^{2}+b_{y}^{2}}},\;Κ={\text{tan}}^{-1}\frac{b_{y}}{b_{z}}$$

As shown in Equations (13)–(15), photogrammetric epipolar resampling is mathematically identical to that under the calibrated approach. The main difference in the calibrated and photogrammetric approaches is that the former calculates relative orientation parameters from left and right extrinsic parameters, which are estimated by calibration patterns, whereas the latter estimates relative orientation parameters directly from tie-points.

The photogrammetric approach may also be sensitive to tie-point noise. In order to overcome the sensitivity problem in epipolar resampling, one may apply the Bayesian approach. The Bayesian approach is a popular estimation technique that uses *a priori* and *a posteriori* error statistics of unknown parameters [35]. In photogrammetric epipolar resampling, one can limit the convergence range of the extrinsic parameters by setting *a priori* error statistics of the parameters. One can also reduce the effects of tie-point errors on the overall estimation by setting *a priori* error statistics of the tie-point measurements. Advantages of the Bayesian approach are that it can make constraints of both the observation equations and the estimation parameters, and that the constraints contribute to the determination of the unknowns as weights. If the *a priori* constraints for unknowns are defined from actual geometric characteristics of the camera arrangement, this approach should work more consistently over stereo image sequences and more robustly over tie-point errors. For many photogrammetric applications, the constraints can be determined from sensors’ specification or operational practice [36,37].

## 4. Datasets and Methodology

For comparative analysis of the epipolar resampling methods, four stereo image sequences (TEST01, TEST02, TEST03, and TEST04) were obtained by two webcams (Microsoft Webcam Cinema). The four image sequences were designed so that error factors were increased from TEST01 to TEST04. Two sequences (TEST01 and TEST02) were obtained in a low resolution mode with an image size of 320 × 240 pixels and two (TEST03 and TEST04) in a high resolution mode with 640 × 480 pixels. Each sequence was obtained with different baseline distances and different camera tilt angles (see Table 1). This was to check the effect of wider baseline and larger tilt angle on epipolar resampling performance. For each sequence, 100 stereo pairs were acquired without camera movement to analyze the consistency of geometry estimation with respect to change of tie-points among different pairs.

**Figure 3:**
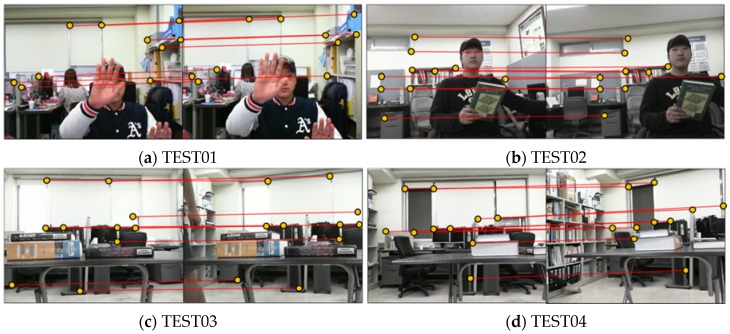
Image datasets used and the 10 tie-points extracted manually for performance evaluation. (**a**) TEST01, (**b**) TEST02, (**c**) TEST03, (**d**) TEST04.

For estimation of epipolar geometry, tie-points were extracted using the scale invariant feature transform (SIFT) algorithm [38] and the outlier removal algorithm [39] based on random sample consensus (RANSAC). Tolerances for outlier removal were set to one pixel for TEST01 and three pixels for the other datasets. The tolerance of one pixel was chosen to set the optimal case, where tie-points had little position error. The tolerance of three pixels was chosen to set the practical case since this value was most widely used in practice. This aims to analyze the robustness against tie-point errors. Additional tie-points were extracted manually at 10 locations from individual image sequences. These points were used to check the accuracy of epipolar resampling. Figure 3 shows one stereo pair from the four image sequences and the 10 tie-points extracted manually.

In experiments, five existing epipolar resampling methods were tested: three developed in computer vision and two in photogrammetry. Table 1 lists out the methods used for the experiments. Note that these methods were chosen as representative methods of the different approaches mentioned in Section 2 and Section 3. “Bouguet” indicates the calibrated approach based on the homographies in Equation (6) developed by Bouguet [40]. “Hartley” is the normalized eight-point algorithm with geometric error minimization based on the Sampson cost function, which is one of the most well-known uncalibrated methods developed in the computer vision field [8]. “Fusiello” is the quasi-Euclidean epipolar rectification developed by Fusiello and Irsara [30]. These two methods belong to the uncalibrated approaches. “Kim” represents a photogrammetric epipolar resampling method based on the relative orientation explained in Section 3 [16]. “Bayesian” is a photogrammetric epipolar resampling with the Bayesian approach for estimating relative orientation parameters, as explained in Section 3.

For the first three methods, we used software available publicly, and for the fourth and fifth methods, we implemented the algorithms in-house. The Bouguet method requires a calibration process determining the intrinsic and extrinsic parameters. For this, 10 pairs of chessboard patterns were used. The intrinsic parameters acquired from this process were also used in the Kim and Bayesian methods. 

As mentioned earlier, the Bayesian method needs to determine *a priori* constraints for estimation parameters and tie-point measurements. These constraints will contribute to correct determination of the unknown parameters by limiting their convergence range in the least-squares sense. We set the *a priori* covariance values for tie-points to one pixel in consideration of the expected rectification error and the expected accuracy of tie-points obtained from the SIFT and outlier removal algorithms. We set the *a priori* covariance values related to orientation parameters to the square of 30° in consideration of the expected variation of camera angles from the ideal case. We set the *a priori* covariance values for the baseline vector ($b_{y}$ and $b_{z}$) to one in consideration of the expected deviation of the baseline vector from the ideal case when assuming $b_{x}=100$. For different experiment settings, different covariance values would be required. For our case, we set relatively large angular covariance values and relatively small positional covariance values. This was because we installed two cameras on a stereo rig and rotated them manually.

A total of four performance measures were used to compare the methods quantitatively. For the evaluation of epipolar geometry, rectification errors ($E_{r}$) and epipolar line fluctuation ($E_{v}$) were used and for the evaluation of image distortion, orthogonality ($E_{o}$) and relative scale change in the result images ($E_{a}$) were used [11]. Rectification errors refer to the residual of *Y* parallaxes in the result image and are measured as the difference of transformed vertical coordinates of tie-points in the left and right images. Epipolar line fluctuation was measured in pixels as standard deviations of the transformed image coordinates over the whole image sequence. In the ideal case, rectification errors and epipolar line fluctuation should be zero. Orthogonality was measured as the angles of the *x* and *y* axes after epipolar resampling, which should be 90° ideally. Relative scale change was measured as the ratio of the two diagonal lines after epipolar resampling, which should be one ideally. For more details regarding performance measures, refer to [11].

## 5. Results of Performance Evaluation

The experiments were carried out by firstly using single pairs and then using image sequences. In the two experiments, the results of the Bouguet method were used as ground truth since they were acquired by calibration patterns. Table 1 summarizes the performance comparison of the five methods applied to single pairs. For the other methods except the Bouguet method, we chose 10 consecutive frames from each image sequence, accumulated tie-points from the 10 frames and estimated a single transformation matrix. Note that due to the limit of the matrix dimension, we chose 10 instead of all frames. Among the four accuracy parameters, epipolar line fluctuation ($E_{v}$) was not included because this is related to an image sequence only. The actual number of tie-points from the 10 frames used for epipolar resampling is also shown in Table 1. In order to better interpret experiment results, distortion measurements were also represented as differences ($\Delta E_{o}$ and $\Delta E_{a}$) with those of the Bouguet method.

In the results of TEST01 and TEST02 datasets, all methods showed satisfactory results in terms of rectification errors. These results demonstrate that although mismatched tie-points are partly included, acceptable transformation accuracy can be produced if the camera configuration is favorable. However, in the case of TEST03 and TEST04 datasets, rectification errors were increased for all test methods. In particular, a large error increase was observed for the uncalibrated epipolar resampling methods, Hartley and Fusiello. This observation agrees well with previous investigations showing that the uncalibrated methods were more prone to tie-point errors in weak stereo geometry [10,11]. The uncalibrated methods also show larger image distortion. This distortion was observed over the whole datasets compared to the results of the Bouguet method. In this experiment, the two photogrammetric methods, Kim and Bayesian, showed almost identical results. It can be interpreted as that effect of tie-point errors was handled better than in the uncalibrated methods by estimating relative orientation parameters directly. A slight rectification error increase for the Kim and Bayesian was observed with TEST04 due to the wide baseline and large tilt angle.

Table 2 summarizes the performance comparison of the epipolar resampling methods applied to all 100 stereo pairs of the four test datasets. Note that the Bouguet was not tried with image sequences since it uses identical homography over all pairs within the same image sequence. For this experiment, we did not accumulate tie-points of consecutive images. We treated each frame independently by estimating a transformation matrix, generating an epipolar resampled pair and calculating the transformation accuracy per frame. Table 2 shows the average number of tie-points used for each image sequence. The accuracy parameters in Table 2 were the average (“Mean” in the Table) and standard deviation (Stdev) of the parameters for the 100 frames.

**Figure 4:**
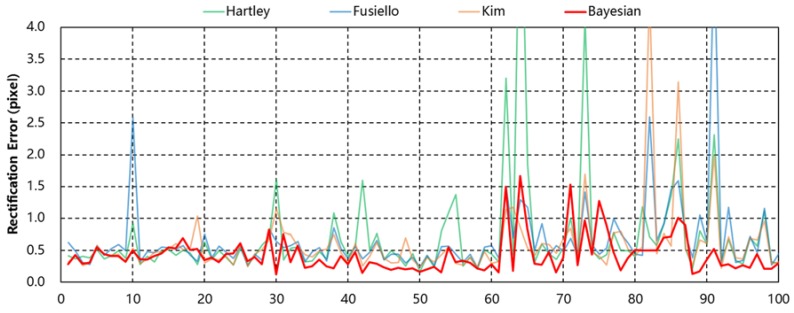
Rectification errors for each frame of TEST02 dataset.

Firstly, we notice that the performance for an image sequence is different from that of single pairs. For a single pair, all methods tested showed small differences among themselves, whereas for image sequences their performance differed significantly. All methods showed variations over image sequences in rectification errors, image distortion errors and epipolar line fluctuation. In particular, epipolar line fluctuation was very significant for all but the Bayesian method for all test datasets. These results imply inconsistent geometry estimation according to the change of the tie-points among each frame and that those methods may not be suitable for handling epipolar resampling of image sequences. Among those tested, the Bayesian method showed the least changes over image sequences. This result demonstrates that by limiting convergence ranges of orientation parameters, stable geometry estimation was achieved. This property of the Bayesian approach may be favorable for image sequences. Figure 4 shows the fluctuation of the rectification errors for all image frames of TEST02 for comparison of the consistency.

Secondly, we can see the tendency of accuracy degradation due to weak stereo geometry, which is similar to the result for the single pair experiment. By comparing TEST01 and TEST02 results, we can check that accuracy has been degraded with larger tie-point errors. Nevertheless, the Bayesian method showed the least degradation. By comparing TEST02 and TEST03, we can observe that the rectification and epipolar line fluctuation errors have been increased with a larger image size and hence with a smaller pixel size in an identical camera. By comparing TEST03 and TEST04, we can observe that the errors have also increased with larger misalignment between two images.

Thirdly, all four errors in Table 2 were increased compared to those of Table 1 for all methods tested. This was anticipated because the number of tie-points used for epipolar resampling in Table 2 was smaller than that in Table 1. However, the Bayesian method produced the least accuracy degradation. This observation may also impose favor to the Bayesian method in case of a smaller number of tie-points.

**Figure 5:**
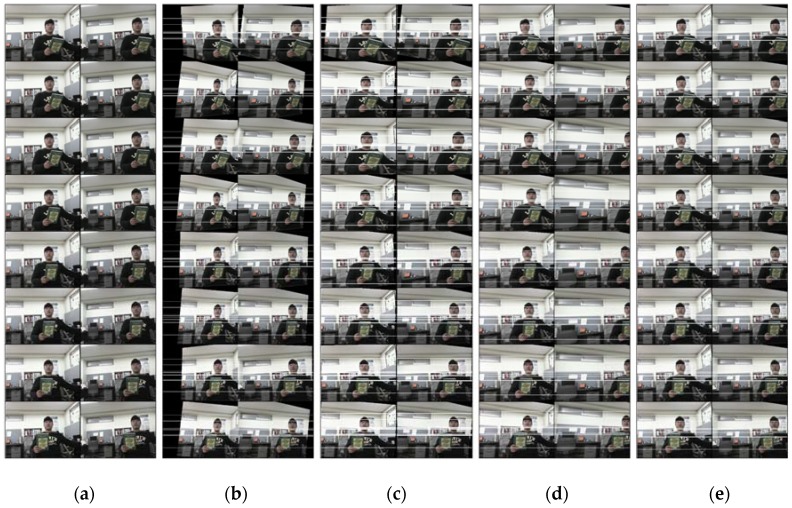
A portion of the results from the consecutive rectified images of TEST02. From left, (**a**) original image and results of the (**b**) Hartley, (**c**) Fusiello, (**d**) Kim, and (**e**) Bayesian methods.

In addition, we checked the performance differences in the methods tested by comparing the resampled image sequences visually. Figure 5 shows a portion of the resampled left and right images of TEST02 from each method. Each column shows the consecutive resampled image pairs from a particular method. The white horizontal lines represent the epipolar lines of the resampled images. We can check the effects of image distortion and inconsistent epipolar geometry estimations in the result images. The resampled sequences with inconsistent epipolar geometry estimations cannot form a stereoscopic image sequence due to abrupt scene changes. The last column showed that the Bayesian method could handle an image sequence with consistency. An explicit comparison of execution time was not carried out because each method tested was under different circumstances. The Hartley and Kim methods were optimized for fast execution (about 32 and 26 frames/s, respectively, on a platform with CPU i5-4460, clock speed 3.20 GHz and memory size 8 GB). The Fusiello and Bayesian methods were not optimized for speed. The Fusiello method was available only in Matlab executables and was very slow (about 2 frames/s). The Bayesian method used very large-sized matrices without efficient matrix decomposition for parameter estimation and was also slow (about 13 frames/s) Reduction of processing time for Fusiello or Bayesian was, however, deemed outside of this paper’s scope and was not tried.

## 6. Conclusions

In this paper, epipolar resampling methods developed in computer vision and photogrammetry were analyzed in terms of rectification errors, image distortion and stability of epipolar geometry. The analysis was focused on the resampling of image sequences.

From the theoretical review, we pointed out that while the epipolar resampling methods developed in two fields are mathematically identical, their performance in parameter estimation may be different.

From the analysis of experiment results, we showed that for epipolar resampling of a single image pair all uncalibrated and photogrammetric methods could be used and, however, that for image sequences all methods tested, except the Bayesian method, showed significant variations in terms of image distortions and epipolar line fluctuation among image pairs within the same sequence. Our results indicate that the Bayesian method is favorable for epipolar resampling of image sequences. These results imply that although differences among the epipolar resampling methods may have been un-noticeable in resampling a single pair, they may not be in resampling image sequences.

As a future study, we will investigate the cause of such variations, ways to minimize them and how to remove remaining small variations in the Bayesian method. Optimization of the processing speed of the Bayesian method will also be studied. Recently, the rapid change of image sources and the diversification of relevant applications are calling for the merge of computer vision and photogrammetric technologies. We hope that our investigation can contribute to the understanding of epipolar resampling technologies developed in both fields and to the development of new stereo applications.

## Figures and Tables

**Table 1:** Results of performance evaluation for single pairs chosen from each image sequence (C, U and P indicate calibrated, uncalibrated and photogrammetric approaches, respectively).

Dataset			Method	$E_{r}$ [pixel]	$E_{o}(\Delta E_{o}$) [°]	$E_{a}$($\Delta E_{a}$)
**TEST01**	Image size	320 × 240	(C) Bouguet	0.52	90.01 (0.00)	1.00 (0.00)
	Baseline width	Narrow	(U) Hartley	0.46	91.77 (1.76)	1.02 (0.02)
	Camera tilt angle	Small	(U) Fusiello	0.48	89.91 (−0.10)	1.00 (0.00)
	Tie-point errors	Small	(P) Kim	0.35	89.99 (−0.02)	1.00 (0.00)
	Tie-points used	858	(P) Bayesian	0.37	90.01 (0.00)	1.00 (0.00)
**TEST02**	Image size	320 × 240	(C) Bouguet	0.64	90.07 (0.00)	1.00 (0.00)
	Baseline width	Narrow	(U) Hartley	0.63	90.73 (0.66)	1.01 (0.01)
	Camera tilt angle	Medium	(U) Fusiello	0.59	90.52 (0.45)	1.01 (0.01)
	Tie-point errors	Large	(P) Kim	0.61	90.09 (0.02)	1.00 (0.00)
	Tie-points used	480	(P) Bayesian	0.43	90.11 (0.04)	1.00 (0.00)
**TEST03**	Image size	640 × 480	(C) Bouguet	0.66	90.07 (0.00)	1.00 (0.00)
	Baseline width	Narrow	(U) Hartley	3.70	89.48 (−0.59)	0.99 (−0.01)
	Camera tilt angle	Medium	(U) Fusiello	3.26	89.98 (−0.09)	1.00 (0.00)
	Tie-point errors	Large	(P) Kim	0.56	90.07 (0.00)	1.00 (0.00)
	Tie-points used	500	(P) Bayesian	0.61	90.06 (−0.01)	1.00 (0.00)
**TEST04**	Image size	640 × 480	(C) Bouguet	0.73	90.38 (0.00)	1.01 (0.00)
	Baseline width	Wide	(U) Hartley	1.51	90.22 (−0.16)	1.00 (−0.01)
	Camera tilt angle	Large	(U) Fusiello	1.26	90.44 (0.06)	1.01 (0.00)
	Tie-point errors	Large	(P) Kim	1.09	90.33 (−0.05)	1.01 (0.00)
	Tie-points used	277	(P) Bayesian	1.13	90.35 (−0.03)	1.01 (0.00)

**Table 2:** Results of performance evaluation for all stereo pairs of the image sequences.

Dataset	Method	$E_{r}$ [pixel]		$E_{o}(\Delta E_{o}$) [°]		$E_{a}$($\Delta E_{a}$)		$E_{v}$ [pixel]
		Mean	Stdev	Mean	Stdev	Mean	Stdev	
**TEST01** Tiepoints used: 98 points/frame	(U) Hartley	0.50	0.14	91.98 (1.97)	0.74	1.02 (0.02)	0.01	3.69
	(U) Fusiello	0.50	0.06	89.90 (−0.11)	0.09	1.00 (0.00)	0.00	4.13
	(P) Kim	0.38	0.05	89.98 (−0.03)	0.02	1.00 (0.00)	0.00	4.44
	(P) Bayesian	0.38	0.02	90.01 (0.00)	0.00	1.00 (0.00)	0.00	0.57
**TEST02** Tiepoints used: 57 points/frame	(U) Hartley	0.71	0.86	89.71 (−0.36)	2.07	1.00 (0.00)	0.06	7.49
	(U) Fusiello	0.66	0.61	90.43 (0.36)	0.74	1.00 (0.00)	0.01	10.59
	(P) Kim	0.60	0.55	90.23 (0.16)	0.10	1.00 (0.00)	0.00	14.05
	(P) Bayesian	0.43	0.29	90.09 (0.02)	0.02	1.00 (0.00)	0.00	0.50
**TEST03** Tiepoints used: 58 points/frame	(U) Hartley	1.58	1.36	91.25 (1.18)	2.18	1.01 (0.01)	0.02	5.45
	(U) Fusiello	1.62	1.50	90.36 (0.29)	0.70	1.00 (0.00)	0.00	17.72
	(P) Kim	1.10	1.24	90.10 (0.03)	0.03	1.00 (0.00)	0.00	5.24
	(P) Bayesian	0.77	0.55	90.07 (0.00)	0.01	1.00 (0.00)	0.00	1.68
**TEST04** Tiepoints used: 29 points/frame	(U) Hartley	1.92	2.24	90.01 (−0.37)	3.44	1.00 (−0.01)	0.02	4.65
	(U) Fusiello	2.09	2.13	90.54 (0.16)	0.29	1.01 (0.00)	0.00	5.84
	(P) Kim	2.66	3.18	90.37 (−0.01)	0.09	1.01 (0.00)	0.00	6.02
	(P) Bayesian	1.63	0.88	90.36 (−0.02)	0.07	1.01 (0.00)	0.00	1.79
